# The Effect of the Menstrual Cycle on Energy Intake: A Systematic Review and Meta-analysis

**DOI:** 10.1093/nutrit/nuae093

**Published:** 2024-07-15

**Authors:** Jessica A L Tucker, Seth F McCarthy, Derek P D Bornath, Jenna S Khoja, Tom J Hazell

**Affiliations:** Department of Kinesiology and Physical Education, Wilfrid Laurier University, Waterloo, ON N2L 3C5, Canada; Department of Kinesiology and Physical Education, Wilfrid Laurier University, Waterloo, ON N2L 3C5, Canada; Department of Kinesiology and Physical Education, Wilfrid Laurier University, Waterloo, ON N2L 3C5, Canada; Department of Kinesiology and Physical Education, Wilfrid Laurier University, Waterloo, ON N2L 3C5, Canada; Department of Kinesiology and Physical Education, Wilfrid Laurier University, Waterloo, ON N2L 3C5, Canada

**Keywords:** estrogen, progesterone, ovarian hormones, food intake, energy balance

## Abstract

**Context:**

Energy intake may differ across the menstrual cycle, with some studies identifying greater energy intake in the luteal phase (LP) compared with the follicular phase (FP) and others finding no clear differences. To date, no study has systematically synthesized the available data to draw more definite conclusions while considering any methodological inconsistencies between studies.

**Objective:**

The aim was to conduct a systematic review/meta-analysis in an effort to determine if there are differences in energy intake between the FP and LP.

**Data Sources:**

A systematic search strategy was developed and the search was conducted in 5 databases for studies that investigated any changes in energy intake across menstrual phases.

**Data Extraction:**

Using Covidence, studies were identified and included if they contained individuals between the ages of 18 and 45 years, maintained an average body mass index (BMI) of 18.5–25 kg/m^2^, had no history of disordered eating, and included energy intake and menstrual cycle measurements in the FP and LP.

**Data Analysis:**

Effect sizes were calculated for each study and a random-effects model was used to pool the results of each study.

**Results:**

Fifteen datasets were included consisting of 330 female participants with a mean age of 26 ± 4 years and mean BMI of 22.4 ± 2.3 kg/m^2^. Overall, there was a statistically significant difference (standardized mean difference = 0.69; P = .039) with increased energy intake in the LP compared with the FP (crude 168 kcal⋅d^−1^ average difference between phases).

**Conclusion:**

Energy intake was found to be greater in the LP compared with the FP, providing insight into the effect of the menstrual cycle on energy intake. However, there were repeated methodological inconsistencies and future work should strive to utilize best practices for both energy intake measurement and menstrual phase specification.

## INTRODUCTION

Exercise interventions directed for weight loss are often not successful in female populations.^1–3^ An influential randomized controlled trial study examined the effects of a 16-month aerobic exercise intervention with an ad libitum diet on 74 young, overweight, or moderately obese (body mass index [BMI] >25.0 kg/m^2^) males (*n* = 31) and females (*n* = 43), where, on average, males lost approximately 5.2 ± 4.7 kg, whereas females gained approximately 0.6 ± 3.8 kg over the 16-month intervention.^4^ Furthermore, a systematic review investigating between-sex differences following dietary interventions reported that males lost more weight (∼4.1–10.8 kg) than females (∼2.9–7.8 kg) in 10 out of 11 studies,^5^ suggesting that males and females may respond to both diet and exercise interventions differently.^6–9^ This variability in weight loss between sexes following weight-loss regimes is possibly due to underlying physiological differences—namely, the menstrual cycle, which involves the cyclic fluctuations of ovarian hormones over a 21-day to 35-day period,^10^^,^^11^ which may influence energy intake.^12^^,^^13^

The typical menstrual cycle is divided into 3 phases—the follicular phase (FP), the ovulatory phase (OP), and the luteal phase (LP)—and each phase is characterized by fluctuations in the ovarian hormones estradiol (E_2_) and progesterone (P_4_).^11^^,^^14–16^ Assuming a 28-day cycle, the FP (menses to approximately day 14) has low E_2_ before rising to its peak concentration in the late FP/early OP, while P_4_ remains low. In the OP (approximately day 14 to day 16; however, variable and best detected using luteinizing hormone [LH] strips that can predict the onset of ovulation), E_2_ begins to decline after reaching its peak and P_4_ continues to remain low. Following the OP is the LP (approximately day 16 to day 28) where E_2_ rises to a secondary peak, although smaller than during OP, and P_4_ rises and reaches its peak concentration (even greater than E_2_) by the mid-LP, before both E_2_ and P_4_ concentrations decrease and menses begins again.^11^^,^^14–16^ Previous research has examined the changes in energy intake in female rats over the estrous cycle and observed a decrease in energy intake associated with the rise in E_2_ (during ovulation). There is also an increase in energy intake during the metestrus/diestrus phase, associated with the rise in both E_2_ and P_4_.^16^^,^^17^ While studies conducted on female rodents provide compelling data on the influence of ovarian hormones on energy intake, there is inconclusive evidence that the same effects occur in human females, and although rodents offer strong similarities in humans in terms of hormonal profile, the estrous cycle only lasts approximately 5 days as opposed to 21–35 days.^15^^,^^17–19^

In humans, several studies demonstrate differences in energy intake across menstrual phases in humans, where energy intake is either greater in the LP compared with the FP^13^^,^^20–26^ or greater in the FP compared with the LP^10^^,^^27^; however, there are also studies demonstrating no changes.^25^^,^^27–31^ These discrepancies may be explained by methodological inconsistencies, particularly for how menstrual phase is characterized.^10^^,^^11^^,^^32–34^ Despite other narrative reviews,^11^^,^^49^^,^^54^ no study has statistically synthesized the available evidence to determine if energy intake is affected by menstrual cycle phase. Therefore, the purpose of this systematic review and meta-analysis is to determine if there are differences in energy intake between the FP and LP while considering the methodological inconsistencies that exist between studies.

## METHODS

The reporting of this systematic review followed the Preferred Reporting Items for Systematic Reviews and Meta-Analyses (PRISMA) guidelines (see Supplementary 2020 PRISMA checklist).^35^ The study selection process was conducted in Covidence systematic review software (Veritas Health Innovation, Melbourne, Victoria, Australia), a screening and data-collection tool. All steps were completed by the authors identified in this manuscript.

### Study selection

Only studies pertaining to energy intake across the menstrual cycle were eligible for inclusion. Studies were included in the systematic review and meta-analysis if they were conducted in participants between the ages of 18 to 45 years to avoid including participants who may be considered peri- or postmenopausal (mean age, 52.8 years).^36^ All studies must have been conducted in biological females who experience an eumenorrheic menstrual cycle (cycle lengths, ∼21–35 days) and included both the FP and LP. All participants were required to be nonsmokers, to have not been diagnosed with a metabolic disorder that could influence the menstrual cycle or energy intake, to have a normal BMI (18–25 kg/m^2^), be recreationally active, and have no indication of a history of disordered eating or relative energy deficiency (RED-S). Exclusion criteria included studies that had individuals who were postmenopausal or were experiencing symptoms of menopause, considered to be underweight or overweight/obese, and/or investigated children or adolescents (<18 years of age). Articles that were not in English, included no title/abstract, were not published in an accredited journal, or did not present the data in numerical form were automatically excluded.

### Search strategy

A comprehensive literature search was conducted in PubMed, Medline, CINAHL, Embase, and Web of Science to identify the dataset of interest using the Population, Exposure, Comparison, Outcomes, and Study design (PECOS) framework (Table 1). To gather all relevant literature regarding energy intake across the menstrual cycle, both free-text and controlled key words were collected under 2 main concepts: (1) energy intake and (2) menstrual cycle. Under “energy intake,” the following key words were collected: “energy intake,” “food intake,” “nutrient intake,” and “calor*.” Under “menstrual cycle,” the following key words were collected: “menstrua*,” “ovarian*,” “luteal,” “follicular,” and “ovulatory.” Key words from each main concept were used in conjunction with one another, using Boolean operators (AND, OR, or NOT), truncation, wildcards, phrase searches, and proximity operators, to refine the searches and complete 1 search across all databases. Included studies must have had participants measure energy intake at least 1 full day within both the FP and LP.

**Table 1.** PECOS Characteristics and Key Words in the Search Strategy for Energy Intake Across the Menstrual Cycle

PECOS guideline	Definition	Key words search
Population	Regularly menstruating females	Menstrua*, ovarian*, ovulatory
Exposure	Any intervention	Luteal
Comparison	Comparator to exposure	Follicular
Outcome	Energy intake across the menstrual cycle	Energy intake, food intake, nutrient intake, calor*
Study design	Any primary studies	Not set

Study selection followed a 2-step process and was independently completed by 2 reviewers (J.A.L.T. and J.S.K.). A blinded third reviewer (D.P.D.B.) settled any discrepancies independently. Titles and abstracts of articles were first screened to filter out studies that were irrelevant and did not relate to the study purpose in the Covidence software. Relevancy was determined individually by the reviewers and each article was considered relevant if the title and abstract discussed the following key words (a process assisted by an automated system within Covidence); (1) follicular, (2) luteal, (3) ovulatory, (4) eumenorrheic, (5) normally menstruating, (6) energy intake, (7) food intake, and (8) dietary intake.

### Assessment of study quality

Three reviewers (J.A.L.T., D.P.D.B., and S.F.M.) independently assessed the methodological quality and risk of bias in each study using a modified tool previously developed and utilized by authors for situations in which tools such as Cochrane’s Risk of Bias tool was not appropriate for their objective.^37^ This modified tool was developed to account for limitations in sample size and inclusion criteria, measurement of outcome variables, and control of confounding variables. All studies included in analysis were identified to be either low, moderate, or high quality in 3 key categories: (1) sample size and inclusion criteria, (2) measurement of outcome, and (3) control for confounders, as described previously.^37^ All 3 reviewers completed the assessment independently, and subsequently met on February 14, 2024, to resolve any discrepancies between them. For each discrepancy, if 2 of the 3 reviewers agreed on a decision (to either include or not include the study), the decision agreed upon by the majority was determined. In the case that all 3 reviewers disagreed, each reviewer presented their individual case and a fourth author (T.J.H.) made the final determination (Table 2). The presence of a sample size calculation and clear statement of inclusion and exclusion criteria was evaluated and each study was determined to be of low (no sample size calculation or statement of inclusion criteria), moderate (no sample size calculation and lack of clarity on inclusion and exclusion criteria), or high (clear statement of sample size calculation and both inclusion and exclusion criteria) quality. For the evaluation of measurement outcomes, each study was evaluated on the presence of efficacious menstrual cycle determination and measurement of energy intake, and each study was determined to be of low (cycle tracking as the only use of menstrual cycle determination as well as reliance on retrospective methods for energy intake measurement), moderate (>1 method of menstrual cycle determination that includes cycle tracking in addition to serum hormone concentrations or the use of LH strips, and the use of retrospective energy intake methodology), or high (>1 method of menstrual cycle determination that includes cycle tracking in addition to serum hormone concentrations or the use of LH strips, and the use of prospective energy intake methodology) quality. For the evaluation of control for confounding variables, each study was evaluated individually on how clearly it controlled for caffeine intake, presence of smokers, medication use, and presence of potential comorbidities and each was assigned a ranking of low (no accountability or control for any of the possible confounding factors), moderate (accounting for 1 or 2 of the listed confounding factors), or high (accounting for all of the listed confounding factors) quality accordingly.

**Table 2.** Quality Assessment Analysis Across All Studies Included in the Analysis

Study, year	Sample size and eligibility criteria	Measurement of outcome	Control for confounders
Barr et al, 1995^43^	Moderate	Moderate	Moderate
Chappell and Hackney, 1997^20^	Moderate	Moderate	High
Cheikh Ismail et al, 2009^19^	Moderate	Moderate	Moderate
Souza et al, 2018^31^	Moderate	Moderate	Moderate
Eck et al, 1997^42^	Moderate	Moderate	High
Elliott et al, 2015^10^	Moderate	Moderate	Moderate
Fong and Kretsch, 1993^29^	Moderate	Moderate	Moderate
Gil et al, 2009^21^	Moderate	High	High
Gong et al, 1989^22^	Low	Moderate	Moderate
Ihalainen et al, 2021^27^	Low	High	Moderate
Johnson et al, 1993^41^	Moderate	High	Moderate
Kammoun et al, 2016^23^	Moderate	Moderate	Moderate
Li et al, 1999^24^	Moderate	Moderate	Low
Lyons et al, 1989^25^	Moderate	Moderate	Moderate
Martini et al, 1994^26^	Moderate	High	High

See Benton et al, 2020^37^. “High” indicates high quality and therefore papers exhibit a low risk of bias; “Moderate” indicates moderate quality and therefore papers exhibit a moderate risk of bias; “Low” indicates low quality and therefore papers exhibit a high risk of bias.

### Data extraction

The following participant characteristics were extracted: mean age (years), mean BMI (kg/m^2^), and mean length of menstrual cycle (days). The following study characteristics were extracted: menstrual cycle definition, approximate days in the cycle when energy intake was measured, number of menstrual cycles studied, which menstrual phases were studied, methods for determining menstrual phase, how long energy intake was tracked, how energy intake was measured, if energy intake was measured consecutively or sporadically in each phase, and absolute energy intake in each phase. The means and SDs for all data were extracted by recording all values directly from each article included in this meta-analysis. Martini et al^26^ provided SEs, which were converted to SDs.

### Statistical analysis

Across studies, energy intake was presented in kilocalories per day (kcal⋅d^−1^), kilojoules per day (kJ⋅d^−1^), or megajoules per day (MJ⋅d^−1^). All means and SDs for energy intake were converted to kilocalories per day (1 kcal = 4.18 kJ = 0.00418 MJ). To improve clinical interpretation, a crude difference in energy intake as kcal⋅d^−1^ was calculated between the phases for each study included in this analysis. Mean energy intakes in the FP and LP were first averaged across the studies, and subsequently the average energy intake in the FP was subtracted from the average energy intake in the LP. It is important to note that this calculation does not account for the varying weights the individual studies received in the actual meta-analysis. Statistical analysis was conducted using R (version 2023.12.1 + 402) in R-Studio (version 2023.12.1 + 402; Posit Software, Boston, United States of America) and we used the following resource^39^ to complete the analysis. Specifically, we used section 3.3.1.3 to calculate the standardized mean difference (SMD) (eqn 1) and the SE (eqn 2) for each study, and section 4.2.1 to guide the meta-analysis. The random-effects meta-analysis was conducted using the *metagen* function from the R package *meta* t.^39^

To calculate SMD, energy intake during the FP ${(\bar{x}}_{FP})\;$was subtracted from energy intake during the LP (${\bar{x}}_{LP})$ and divided by the SD during the FP (SD_*FP*_). Harrer et al^39^ suggests using the SD from the initial test (assuming a pre-post design) when conducting a within-subject analysis; however, as not all studies had participants complete the sessions in the same order, we used the FP data for all calculations. The average difference in SD between phases was only 10 kcal and when SMD was calculated with SD_LP_ and the model run with these data, the overall effect was still statistically significant (data not shown).


(1) $$\mathrm{SMD}=\;\frac{{\overset{-}{x}}_{\mathrm{LP}}-{\overset{-}{x}}_{\mathrm{FP}}}{{\mathrm{SD}}_{FP}^{}}$$

To calculate the SE (eqn 2) the SMD value (SMD; previously calculated), along with the number of participants (*n*) and the correlation between the 2 phases were used. As no published research had correlated energy intake during the FP and LP, and none of the corresponding authors of the studies included in this manuscript returned any e-mails regarding correlation values from their respective studies, a conservative *r* value was used (0.2, low correlation) for all SE calculations.


(2) $$\mathrm{SE}\;=\;\sqrt{\frac{2(1-r_{t1t2})}{n}+\frac{{\mathrm{SMD}}^{2}}{2n}}$$

## RESULTS

### Study selection

**Figure 1.**
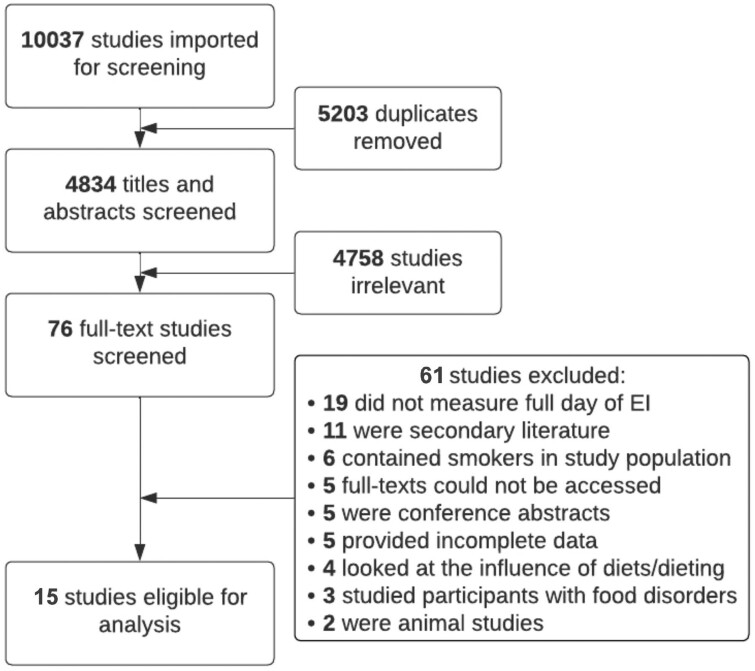
Flowchart of the Study Selection Process and Studies Included in the Meta-Analysis. Abbreviation: EI, energy intake

A comprehensive literature search was conducted in PubMed, Medline, CINAHL, Embase, and Web of Science on January 6, 2023, and a second up-to-date search took place on January 23, 2023^40^ (Figure 1). From this search, a total of 10 037 articles were imported into Covidence. From this, 5203 articles were removed as they were automatically characterized as duplicates. Titles and abstracts of 4834 articles were first screened to filter out studies that were irrelevant and did not relate to the study purpose in the Covidence software. Seventy-six studies passed the title and abstract screening, and the full-text manuscripts were downloaded directly from Covidence and reviewed to determine if the eligibility criteria were met. Of the 76 studies that were included in full-text screening, 19 studies did not measure energy intake for a full day, 11 were considered to be a secondary analysis, 6 contained smokers in the study population, 5 full-texts could not be completely accessed, 5 were conference abstracts and not published papers, 5 provided incomplete datasets, 5 looked at the influence of diets/dieting only, 3 studied participants with food-related disorders, and 2 were animal studies. A total of 15 studies passed both stages of screening and were extracted and included in the final analysis.

### Study and participant characteristics

The 15 datasets included in this meta-analysis consisted of 330 female participants (Table 3) with a mean age of 26 ± 4 years (range, 20–36 years) and mean BMI of 22.4 ± 2.3 kg/m^2^ (range, 18.7–26.9 kg/m^2^). All studies, with the exception of 2, recruited participants between the ages of 20 and 30 years.^22^^,^^41^ Only 4 studies clearly identified that the population recruited was recreationally active,^20^^,^^26^^,^^27^^,^^41^ while the other studies provided no definition of physical activity status. Four studies included in this analysis involved different datasets or groups within the study, which remained separated, and these specific datasets were not used.^20^^,^^27^^,^^42^ One of these studies involved the comparison of physically active females to physically inactive females^20^ and 2 studies compared users of monophasic hormonal birth control with those who were not.^27^^,^^42^

**Table 3.** Study Characteristics Outlining Participant Characteristics and Methodology for Energy Intake and Menstrual Cycle

Study, year	Age (y) and BMI (kg/m^2^)	MC length (d)	MC definition		Cycles studied	Method	Energy intake		No. of days EI measured (d·phase)	Average EI across each phase, kcal·d^-1^ (kJ·d^-1^)	
			FP	LP			FP	LP		FP	LP
Barr et al, 1995^43^	28 ± 5	NA	NA	NA	3	Temp	Day 3-8	Day 20-26	3	1915 ± 565	2216 ± 642
	21.8 ± 2.1									(8012 ± 2363)	(9271 ± 2686)
Chappell and Hackney, 2007^20^	22 ± 1	30 ± 2	NA	NA	1	Temp	Day 10	Day 20	10	1901 ± 124	2037 ± 150
	21.3									(7953 ± 518)	(8522 ± 627)
Cheikh Ismail et al, 2009^19^	22 ± 3	29 ± 2	Day 0-10	Day 20–menses	1	Cycle tracking	NA	NA	2	1255 ± 526	1363 ± 550
	22.2 ± 3.9									(5250 ± 2200)	(5702 ± 2301)
Eck et al, 1997^42^	20 ± 3	27 ± 2	Day 0-13	Day 15–menses	2	LH kits	Day 7-9	Day 22-24	Daily	1601 ± 359	1769 ± 359
	20.8 ± 3									(6698 ± 1502)	(7401 ± 1502)
Elliott et al, 2015^10^	24 ± 1	30 ± 6	25-49%^a^	51-100%^a^	1 or 2	Cycle tracking	NA	NA	3	1663 ± 413	1384 ± 307
	20.2 ± 2.8									(6957 ± 1727)	(5790 ± 1284)
Fong and Kretsch, 1993^29^	28 ± 4	26 ± 2	Day 4-11	Day 16-28	1	Temp	NA	NA	Daily	2360 ± 543	2501 ± 539
	22.4 ± 2.0									(9874 ± 2271)	(10464 ± 2255)
Gil et al, 2009^21^	28 ± 4	NA	Day 5-9	Day 20-28	1	[E_2_/P_4_]	NA	NA	3	1705 ± 251	2227 ± 370
	22.20 ± 2.0									(7133 ± 1050)	(9317 ± 1548)
Gong et al, 1989^22^	31 ± 7	27 ± 2	Day 6-14	Day 15-18	1	Temp	Day 5-13	Day 15-28	Daily	1833 ± 146	2040 ± 156
	22.4									(7669 ± 610)	(8535 ± 652)
Ihalainen et al, 2021^27^	26 ± 4	NA	NA	NA	1	LH kits and [E_2_/P_4_]	Day 7-11	7 Days post-LH surge	3	2340 ± 540	2270 ± 370
	24.0									(9790 ± 2259)	(2497 ± 1548)
Johnson et al, 1993^41^	32 ± 4	28 ± 5	Rising E_2_, low P_4_	Peak P_4_, increase in E_2_	1	Temp and [E_2_/P_4_]	NA	NA	Daily	1711 ± 421	1875 ± 445
	21.5									(7158 ± 1761)	(7845 ± 1861)
Kammoun et al, 2016^23^	27 ± 8	NA	NA	NA	1	Cycle tracking	Day 1-3	Day 17-25	1	1688 ± 332	2164 ± 322
	25.6 ± 5.2									(7062 ± 1389)	(9054 ± 1347)
Li et al, 1999^24^	21 ± 1	31 ± 2	Day 0-10	6-10 Days post-LH surge	1	LH kits	NA	NA	3	1457 ± 281	1668 ± 441
	19.6 ± 1.4									(6096 ± 1175)	(7062 ± 1845)
Lyons et al, 1989^25^	24	28 ± 1	Day 5 – +ve LH test	4 Days post-LH surge	1	LH kits	NA	NA	Daily	2012 ± 69	2150 ± 86
	22.8									(8418 ± 288)	(8995 ± 359)
Martini et al, 1994^26^	27 ± 4	NA	Day 0-9	7-9 Days post-LH surge	4, 5, or 6	LH kits, [E_2_/P_4_], temp	Day 7-9	7-9 Days post-LH surge	3	1749 ± 37	1908 ± 38
	22.1 ± 1.9									(7319 ± 154)	(7983 ± 158)
Souza et al, 2018^31^	22 ± 1	NA	NA	NA	1	Cycle tracking	Day 5-9	Day 20-25	1	1694 ± 437	1738 ± 414
	23.4 ± 0.9									(7087 ± 1828)	(7271 ± 1732)
Pooled characteristics	26 ± 4	29 ± 2								1791 ± 307	1959 ± 346
	22.4 ± 2.3									(7494 ± 1284)	(8196 ± 1445)

a Represents percentage of cycle length for determining menstrual phase.

Abbreviations: BMI, body mass index; E_2_, estradiol; [E_2_/P_4_], serum E_2_/P_4_ concentrations; EI, energy intake; FP, follicular phase; LH, luteinizing hormone; LP, luteal phase; MC, menstrual cycle; NA, not available; P_4_, progesterone; Temp, temperature; +ve, positive.

### Study quality

The assessment of study quality is shown in Table 2. For the evaluation of sample size and inclusion and exclusion criteria, all studies but 2 were considered as having moderate quality,^22^^,^^27^ where neither a sample size calculation nor clear indications of inclusion and exclusion criteria were present. For measurement outcomes, all studies were considered of moderate quality with the exception of 4 studies,^21^^,^^26^^,^^27^^,^^41^ which exhibited high-quality measurements of energy intake and menstrual cycle estimation. Finally, all studies but 4 were considered high quality in terms of controlling for possible confounding variables,^20^^,^^21^^,^^26^^,^^42^ and 1 study was considered to be low quality^24^ for a lack of addressing possible confounders directly.

### Difference in energy intake between menstrual cycle phases

**Figure 2.**
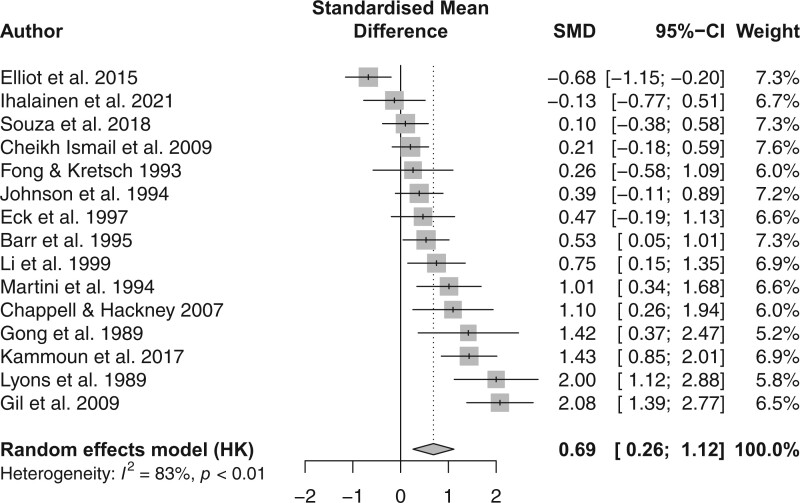
Forest Plot of Individual Effect Sizes (Calculated Using SMD), Weighted Percentage, and Overall Effect Size Comparing the Difference in Energy Intake Between the Follicular Phase and Luteal Phase. Positive values favor greater energy intake in the LP compared with the FP. Abbreviations: FP, follicular phase; HK, Knapp-Hartung adjustment; LP, luteal phase; SMD, standardized mean difference

The overall effect size was moderate (SMD = 0.69, *P *= .039, *n* = 330), and the interstudy heterogeneity was estimated to be τ^2^ = 0.4972, with an *I*^2^ = 83% (*P *< .01).^38^ A forest plot (Figure 2) presenting the differences in energy intake between the FP and LP was generated. An overview of the energy intake data in individual studies is presented in Table 3. This plot includes each individual study, the corresponding effect sizes calculated using SMD, the weighted percentage, and the overall effect size. The results identified an average increase in energy intake in the LP compared with the FP, with an average difference of 168 kcal⋅d^−1^.

## DISCUSSION

While the impact of menstrual cycle phase on energy intake has been explored previously,^10^^,^^20–22^^,^^24–27^^,^^29^^,^^31^^,^^41–43^ these studies demonstrated conflicting results and have yet to be synthesized. Therefore, this systematic review and meta-analysis investigated if differences in energy intake exist across the menstrual cycle phases that represent the majority of the menstrual cycle.^15^ Energy intake was higher in the LP compared with the FP (*P *= .39, SMD = 0.69) (Figure 2), with an increase in energy intake in the LP compared with the FP by 168 kcal⋅d^−1^. Importantly, there was a large degree of heterogeneity in the measurement of energy intake as well as menstrual phase determination, although this is to be expected as female-specific research has evolved greatly over the last 4 decades and methodological designs have improved greatly.^15^

Of the 15 datasets included in analysis, 8 reported statistical differences in energy intake between the LP and FP^10^^,^^20–24^^,^^25^^,^^26^ and 7 reported no differences.^19^^,^^27^^,^^29^^,^^31^^,^^41–43^ Certain studies had multiple datasets to include those with oral contraceptives,^27^^,^^42^ while 1 study had 2 groups in which 1 was considered to be physically inactive^20^; however, only the groups that were not taking oral contraceptives and were characterized specifically as being recreationally active were used for analysis. Of the 7 studies that reported no statistically significant differences, 6 reported effect sizes that favored an increase in energy intake in the LP compared with the FP (average, ∼99 kcal⋅d^−1^).^19^^,^^29^^,^^31^^,^^41–43^ Of the 8 studies that identified differences in energy intake between FP and LP, 7 demonstrated greater energy intake (average, ∼275 kcal⋅d^−1^) in the LP compared with the FP.^20–24^^,^^25^^,^^26^ Interestingly, only 1 study actually demonstrated greater energy intake in the FP compared with the LP,^10^ with a difference of approximately 260 kcal⋅d^−1^. There is no apparent physiological explanation to support greater energy intake in the FP compared with the LP for this particular study. The discrepancy between these studies could likely be due to the result of methodological differences. This particular study identified a greater energy intake in the FP and used a percentage of cycle length completed to determine the phase in which menstruation marked 0% and the FP and LP were 25–49% and 50–100%, respectively. Menstrual phase was not confirmed via LH strips or blood sampling,^10^ which has now been identified as a critical step in accurate menstrual phase characterization.^15^ This meta-analysis as well as the majority of research support greater energy intake in the LP compared with the FP, which is line with both murine model data as well as the hypothesis that a greater energy intake in the LP is a biological response to support a potential pregnancy, by ensuring that females are not in an energy deficit during a period of high energy demand.^45–47^ There was large variability in the determination of menstrual phase across all studies due to inconsistencies in defining and verifying the menstrual cycle phases as well as the timing of energy intake measurement throughout the menstrual cycle.^15^ It is important to note that only 3 studies used more than 1 method of menstrual phase verification, which is now considered to be insufficient in any current menstrual cycle research.^15^

It has been speculated that E_2_ plays a role in energy intake through mechanisms related to appetite.^13^^,^^48^^,^^49^ E_2_ receptors (ERs) are found as 2 subtypes: ERα and ERβ,^50^ where ERα is the key E_2_ receptor in the brain and is highly expressed on neurons within the arcuate nucleus,^51^ more specifically pro-opiomelanocortin (POMC), which triggers a signaling cascade to alter appetite and subsequent energy intake.^50^ In rodents, as E_2_ increases in the late FP towards its peak concentrations in the OP with no increase in P_4_, POMC activity is increased and a resulting decrease in energy intake during the late FP/OP occurs.^52^ Previous work in female rats has demonstrated that removal of ERα receptors results in increases in energy intake and the presence of active ERα in the brainstem reduces energy intake,^53^^,^^54^ further supporting that E_2_ may bind to appetite-inhibiting neurons in the brain, which could explain the lower energy intake in the OP where a greater concentration of E_2_ is present. It is important to note that, due to the lack of available energy intake data, the OP was excluded from this analysis, which highlights the need for future research including this short, but important, phase of the menstrual cycle.

Although the role of P_4_ in appetite regulation is less understood,^55^^,^^56^ mice supplemented with physiological doses of endogenous P_4_ had increased 24-hour energy intake compared with those supplemented with E_2_ only or a combination E_2_ and P_4_.^57^ These results suggest that P_4_ may have appetite-stimulating properties, subsequently increasing energy intake, or has inhibitory effects on appetite-inhibiting properties of E_2_, possibly through antagonistic ERα activity.^16^^,^^55^^,^^58^ It is important to note that, although rats are the animal model most often used to compare female hormonal fluctuations, the rat estrous cycle only lasts 4 to 5 days, has hormone fluctuations characterized by 4 distinct phases as opposed to 3, and female rats do not experience menstruation.^44^ These key differences between female rats and humans are important to consider as they impact the mechanistic interaction of ovarian hormones and neuronal activity related to appetite.^15^ In the estrous cycle, rats experience a similar rise and peak of E_2_ and P_4_ in the proestrus phase of the estrous cycle, which is considered the first stage and only lasts approximately 14 hours,^59^ reflecting similar hormone fluctuations in the LP in humans that lasts for approximately 14 days. However, rats do not experience a phase in which E_2_ peaks independently of P_4_,^59^ highlighting the necessity for human research to understand the role of the ovarian hormones in appetite regulation and further supporting the need for the current study as well as future research on the role of ovarian hormones in appetite and energy intake research in females.

Energy intake was measured in all studies included for analysis via self-reported dietary records, which have previously been reported to be subject to bias,^60^ despite remaining the most common method for measuring energy intake.^51^ Four of these studies attempted to improve validity of the food logs by weighing the foods consumed by each participant,^19^^,^^22^^,^^25^^,^^43^ 1 study provided a “menu” of food products from participants to choose from,^29^ and only 1 study clearly defined that a registered dietitian had worked with participants to confirm energy intake.^23^ These complexities highlight that the comparison of energy intake data validity between studies is difficult, especially in conjunction with inconsistent menstrual phase characterization. Recent methodological advances using imagery-based smartphone applications, particularly those monitored by a registered dietitian,^61^ may be helpful in ensuring energy intake data are consistent and reliable.^62–64^ Eight of the studies included in this analysis asked participants to log their food on consecutive days,^20^^,^^22^^,^^25^^,^^26^^,^^29^^,^^41–43^ while 3 studies did not state whether or not food logging was done on consecutive days or was completed sporadically.^19^^,^^23^^,^^31^ From the 5 studies that measured energy intake daily, 3 studies found a greater energy intake in the LP compared with the FP^22^^,^^41^^,^^42^ and 2 found no differences,^25^^,^^29^ indicating that the period of time for which energy intake is measured may not be a predictor of energy intake across the menstrual cycle.^15^ Three of these studies that either found no differences between menstrual phases or greater energy intake in the FP compared with the LP also had significant individual variability in energy intake of up to approximately 945 calories per day^10^^,^^29^^,^^31^ and 1 of these studies was underpowered.^29^ In addition, 4 of these studies did not provide or report clearly the approximate day of testing phase for all phases.^10^^,^^25^^,^^29^^,^^31^

While this systematic review and meta-analysis is the first to systematically synthesize the available data on energy intake across the menstrual cycle, there are several limitations to acknowledge. First, participants who completed at home dietary records may have over- or underestimated their energy intakes, which may have an influence on the mean differences between the FP and the LP.^65^^,^^66^ Second, it is worth considering that energy intake may not be uniform across the week, as food consumption collected over the weekends compared with weekdays may differ^24^ and this was not accounted for in many of the studies included in analysis. Third, it is important to consider that the follicular phase contains a hormonal profile where E_2_ and P_4_ are low for most of the phase; however, towards ovulation, E_2_ begins to increase. Because of the lack of clarity within some studies on how exactly menstrual phase is determined and taking into consideration interindividual variability, it remains difficult to ensure that all participants maintained a low hormonal profile in the FP. Fourth, there is a possibility that this meta-analysis is unable to account for data that were collected but not published and we cannot completely exclude the possibility of publication bias. Finally, the current review includes literature that dates back as early as 1988, and due to the less detailed standard of menstrual phase determination in prior years, many technical flaws are present in these studies.^15^ With the recent emphasis on the inclusion of females in research studies, it is important that researchers account for hormonal patterns related to the menstrual cycle and apply effective methods for determining menstrual phase. Considering the small quantity of research pertaining to the inclusion criteria, no study was excluded based on date of publication.

## CONCLUSION

In conclusion, this systematic review and meta-analysis determined that energy intake is greater in the LP compared with the FP, with a crude difference of approximately 168 kcal⋅d^−1^. The overall effect size for differences in energy intake between phases moderately favors the LP having a higher energy intake, which may be attributed to the presence of elevated ovarian hormones during the LP and supports similar findings found in animal studies,^53–55^ and is possibly explained by the need for females to maintain a neutral/positive energy balance in the LP in order to support a potential pregnancy.^45–47^ There are methodological inconsistencies across the existing literature pertaining to both how menstrual phase is characterized and measurement of energy intake, suggesting that future work using robust methods to identify the menstrual cycle phase and best methods for the determination of energy intake is warranted. This study provides valuable insight on the effect of the menstrual cycle on energy intake, supports previous data using animal models, and further highlights the need for more rigorous research focused on female-specific physiology in hopes of improving the understanding of the menstrual cycle, specifically the fluctuations of ovarian hormones, and potential effects on energy intake.

## Supplementary Material

nuae093_Supplementary_Data

## Data Availability

All data analyzed in this review as well as searching protocol can be made available upon request. This review was not registered.
